# Retention of knowledge and skills after Emergency Obstetric Care training: A multi-country longitudinal study

**DOI:** 10.1371/journal.pone.0203606

**Published:** 2018-10-04

**Authors:** Charles A. Ameh, Sarah White, Fiona Dickinson, Mselenge Mdegela, Barbara Madaj, Nynke van den Broek

**Affiliations:** Centre for Maternal and Newborn Health, Liverpool School of Tropical Medicine, Liverpool, United Kingdom; Monash University, AUSTRALIA

## Abstract

**Objective:**

To determine retention of knowledge and skills after standardised “skills and drills” training in Emergency Obstetric Care.

**Design:**

Longitudinal cohort study.

**Setting:**

Ghana, Malawi, Nigeria, Kenya, Tanzania and Sierra Leone.

**Population:**

609 maternity care providers, of whom 455 were nurse/midwives (NMWs)

**Methods:**

Knowledge and skills assessed before and after training, and, at 3, 6, 9 and 12 months. Analysis of variance to explore differences in scores by country and level of healthcare facility for each cadre. Mixed effects regression analysis to account for potential explanatory factors including; facility type, years of experience providing maternity care, months since training and number of repeat assessments.

**Main outcome measures:**

Change in knowledge and skills.

**Results:**

Before training the overall mean (SD) score for skills was 48.8% (11.6%) and 65.6% (10.7%). for knowledge. After training the mean (95% CI) relative improvement in knowledge was 30.8% (29.1% - 32.6%) and 59.8% (58.6%– 60.9%) for skills. Mean scores for knowledge and skills at each subsequent assessment remained between those immediately post-training and those at 3 months. NMWs who attended all four assessments demonstrated statistically better retention of skills (14.9%, 95% CI 7.8%, 22.0% p<0.001) but not knowledge (8.6%, 95% CI -0.3%, 17.4%. p = 0.06) compared to those who attended one or two assessments only. Health care facility level or experience were not determinants of retention.

**Conclusions:**

After training, healthcare providers retain knowledge and skills for up to 12 months. This effect can likely be enhanced by short repeat skills-training sessions, or, ‘fire drills’.

## Introduction

Direct maternal deaths account for most of the 300 000 maternal deaths which occur globally each year and are the leading cause of death in low- and middle-income settings (LMIC). Emergency Obstetric Care (EmOC) comprises of an internationally agreed care package used to treat the most frequently occurring complications (haemorrhage, (pre)eclampsia, sepsis, incomplete miscarriage or unsafe abortion and obstructed labour) which if unrecognised and untreated result in maternal death. (**Table 1**) It is estimated that up to 15% of pregnant women may develop obstetric complications which require EmOC [1,2].

**Table 1:** Components or signal functions for the Emergency Obstetric Care (EmOC) package.

Basic EmOC services	Comprehensive EmOC services
1. iv/im antibiotics	All included in Basic EmOC (1–7) plus:
2. iv/im oxytocic drugs	8. Caesarean Section
3. iv/im anticonvulsants	9. Blood Transfusion
4. Manual removal of placenta	
5. Removal of retained products of conception (e.g. by manual vacuum aspiration)	
6. Assisted vaginal delivery (e.g. ventouse delivery)	
7. Resuscitation of the newborn baby using a bag and mask	

Source: WHO 2009: Managing emergency obstetric care: a handbook [2]

For every 500,000 population, at least 5 healthcare facilities should be in place which can provide EmOC 24/7. These should be geographically equitably distributed to ensure all women have access to these services if needed [2]. In a systematic review by Nyamtema et al (2011), EmOC training was one of the interventions implemented in 52–65% of 54 maternal and newborn health (MNH) programmes which had contributed to a significant reduction in maternal mortality [3]. Previous studies evaluating the change in knowledge and skills immediately after EmOC training, showed significant improvement for all cadres providing maternity care in low and middle-income countries [4,5]. Teaching responsibilities, previous in-service training and a high percentage of time spent providing maternity care are associated with better knowledge and skills [5]. EmOC training is a large component of many Maternal and Newborn Health intervention programmes, and, considered mandatory training in some settings. However, only a few studies have assessed whether improved competence demonstrated immediately after training, is retained over time.

Two studies evaluated retention of knowledge and skills after in-service training that included all components of EmOC. A randomised controlled trial including 36 health care providers conducted in Pakistan reported knowledge was retained at 6 months [6] while Tang et al (2016) in a before after study (n = 134) reported retention of knowledge and skills at 3 months but not at 6 months post training [7]. None of these studies accounted for potential confounding factors.

A standardised 'skills and drills' package training including all of the EmOC components, was provided to healthcare providers in six countries in sub-Saharan Africa. As part of the evaluation of effectiveness of training, the retention of knowledge and skills was assessed quarterly for up to 12 months following the training. In addition, factors determining retention of knowledge and skills were explored.

## Methods

The STrengthening the Reporting of OBservational studies in Epidemiology (STROBE) guidelines were used to report the findings of this longitudinal cohort study. Maternity care providers working in healthcare facilities designated to provide Basic or Comprehensive EmOC located in two districts/regions in each of six countries (Ghana, Kenya, Malawi, Nigeria, Sierra Leone and Tanzania) were trained between May and November 2013 using a standardized 'skills and drills' EmOC training package developed in 2006 by Centre for Maternal and Newborn Health (CMNH) at the Liverpool School of Tropical Medicine (LSTM) in partnership with the World Health Organization (WHO) and the Royal College of Obstetricians and Gynaecologists (RCOG). The training content includes all the components and signal functions of EmOC. (**Table 1**) Healthcare providers are trained to recognize and manage women with the main complications leading to maternal death including; postpartum and ante-partum haemorrhage, (pre)-eclampsia, sepsis, complications of obstructed labour and abortion as well as management of breech delivery, cord prolapse, twin delivery, shoulder dystocia and early newborn care. [8]

### Study participants

All cadres providing maternity care in each country and setting were eligible and included: medical doctors, nurse-midwives (NMW), mid-level cadre (Clinical Officers (CO) and Medical Assistants (MA)), and junior cadre (Maternal and Child Health Aides (MCHA) and Community Health Extension Workers (CHEW)). Only NMWs were represented in all study countries. The various cadre providing maternity care in SSA have been described elsewhere. [9]

### Sample size

The minimum increase in the score for knowledge or skills that was considered clinically important was 20%. [10,11] A sample size of 72 would have 90% power to detect a change of 20% between pre-and post-training scores, assuming a standard deviation of 20 for these changes. A sample size of 585 maternity care providers (MCP) was determined, this included 25% allowance for any loss during follow-up.

### Assessment of knowledge and skills

Participants were assessed immediately before and after training, and, subsequently again at 3, 6, 9 and 12 months after training. Tests were anonymized, but each participant was allocated a unique identifier so that pre-and post-training assessments could be linked. A questionnaire was completed at each assessment to collect information on gender, level of healthcare facility (designated to provide Basic or Comprehensive EmOC), years of experience, area of work (maternity or other) and were asked to report on any other relevant training that had been completed in the interim period **S1 Appendix: KSRS Questionnaire**. Knowledge and skills assessment questions were pre-tested and randomly selected from a question bank (322 knowledge, 15 scenarios for skills testing). Each participant was required to complete 40 multiple-choice knowledge questions, and eight scenario-based skills tests using obstetric/newborn mannequins over a period of 80 minutes. The same tests were administered immediately before and after the training, but, for each subsequent assessment, a new set of questions was randomly selected from the question bank **S2 Appendix: Knowledge and Skills Tests**.

All assessors were trained using the same protocol. During each skills assessment, the relevant assessor noted any skills that were not correctly performed, and, provided feedback after the healthcare provider had completed the assessment and this had been scored **S3 Appendix: AVD Practical Test**.

### Analysis

For each participant, separate knowledge and skills scores were derived for each assessment when there was no missing data for that score to be derived. Scores for each assessment were expressed as a percentage.

To examine each participant’s relative change in scores over time, their pre-training score (*pre*) was used as the reference value to determine the maximum possible score change (100 minus *pre*). Scores achieved subsequently (*m*) were used to calculate the percentage of the maximum possible change in score attained at that assessment point, referred to as the relative change / improvement (relative increase = (*m-pre)*/(100-*pre); relative decrease =* (*m-pre)*/*pre*).

The assessment visit number was defined as the visit number for each individual participant: the first post-training assessment (whether it was at month 3, 6, 9 or 12) was assessment 1. Similarly, if a participant attended all four assessments, their assessment number at month 12 was 4. Thus, this variable indicates the cumulative number of repeat assessments completed by the participant.

For pre-training knowledge and for pre-training skills scores separate analyses of variance were performed for each cadre to determine if there were differences by facility type, and/or country. A *p* value of <0.05 was considered statistically significant.

For each of knowledge and skills the relative change data for NMWs from all countries and assessment rounds was analysed using mixed effects regression analysis, to account for the following variables: month (relative to training month: 0 (post training), 3, 6, 9 or 12); assessment number; potential confounding variables (i.e. facility level and years of experience (<2 years, 2-<4 years, 4-<7 years, 7-<13 years and 13 years or longer). Each of these variables was defined as a factor rather than a covariate as either the variable was binary or it was not assumed that the effects would be linear. The relevant pre-test scores were used as a covariate in each analysis. Random subject intercept and slope terms for month were also included to account for variation between respondents. To examine evidence of heterogeneity between countries the interaction between country and assessment number was included. When there was evidence of heterogeneity between countries each country was also analysed separately. Likelihood ratio tests were used to assess if the explanatory variables were significant, and only variables which were statistically significant were retained in the model. Estimates reported were derived using restricted maximum likelihood estimation.

To compare relative change scores for NMWs at 12 months between those who attended all for assessments and those who only completed one or two of the follow up assessments two sample t tests were used.

## Results

In total, 609 healthcare providers participated in the study and were assessed at least once after the training. The total loss to follow-up rate was 25.3% (3-6M: 5.3%, 6-9M: 7.2%, 9-12M: 12.8%). The 12-month assessment was attended by 75% (455) of all participants.

### Characteristics of participants

The majority (75% (455)) of the participants were nurses-midwives, 2.3% (14) were medical doctors, 14% (85) were mid-level staff and 9% (55) were junior cadre staff **Table 2**. All medical doctors worked at a healthcare facility designated to provide Comprehensive EmOC. Nurse/midwives worked at both (BEmOC (43.7%) and CEmOC (56.3%)), while 98% of junior cadre participants worked at a healthcare facility designated to provide Basic EmOC.

**Table 2:** Characteristics of participants.

Variable/ Category	Medical Doctors	Mid-level cadre	Nurse-midwives	Junior cadre	Total
Total	14	85	455	55	609
**Country**					
**Ghana**	**11**		**114**		**125**
**Kenya**	**3**	**18**	**97**		**118**
**Malawi**		**30**	**63**	**23**	**116**
**Nigeria**			**62**	**32**	**94**
**Sierra Leone**			**64**		**64**
**Tanzania**		**37**	**55**		**92**
**Gender**					
**Male**	**9**	**58**	**59**	**10**	**136**
**Female**	**2**	**27**	**394**	**45**	**468**
**NR**	**3**		**2**		
**Number of years of experience**					
**<2**	**10**	**29**	**99**	**8**	**146**
**2-<4**	**3**	**19**	**77**	**13**	**111**
**4-<7**	**1**	**15**	**85**	**12**	**113**
**7-<13**	**0**	**7**	**94**	**8**	**109**
**13 or more**	**0**	**11**	**68**	**1**	**80**
**NR**		**4**	**41**	**13**	
**Level of service designated to provide**					
**Basic EmOC**	**0**	**37**	**199**	**54**	**290**
**Comprehensive EmOC**	**14**	**41**	**217**	**1**	**273**
**Number of healthcare providers assessed**					
**Month 3**	**9**	**72**	**366**	**42**	**489**
**Month 6**	**6**	**73**	**349**	**42**	**470**
**Month 9**	**3**	**71**	**368**	**44**	**487**
**Month 12**	**6**	**64**	**343**	**42**	**455**

EmOC = Emergency Obstetric Care

Just over one in five (22%) participants had less than two years of experience providing maternity care, including 46.4% of medical doctors and 21.8% of nurse-midwives. In contrast, 13% had more than 13 years of experience providing maternity care. There was no significant change in area of work (redeployment from maternity wards) for participants before compared to after the training.

### Pre-training knowledge and skills

Before training the overall mean scores for skills was lower than the scores for knowledge; 48.8% (SD 11.6%) vs 65.6% (SD 10.7%) respectively. Analysis of variance using these scores found a significant difference between countries for both knowledge and skills for NMW and mid-level cadre only. Mean knowledge scores in Tanzania were lowest (58.1% for NMWs and 63.2% for mid-level staff), and, highest in Kenya and Malawi (67.7% and 70.5% respectively). For skills, for both these cadres mean scores were highest (59.9% - 60.6%) in Malawi and lowest among NMWs in Nigeria (42.8%) and mid-level staff in Kenya (46.0%). Overall, junior cadre staff had significantly different scores for skills (not knowledge) with the mean for Nigeria (30.7%) lower than for Sierra Leone (40.6%). The only difference regarding facility level was for knowledge among mid-level cadres, with those working in CEmOC facilities obtaining mean scores that were 6.3% higher than those in BEmOC facilities.

### Change in knowledge and skills

After training the mean score for knowledge was 76.6% (SD 9.1%) and the mean score for skills was 79.2% (SD 9.1%). Overall, there were statistically significantly improved scores for both knowledge and skills for all cadres immediately after the training. The mean (95% CI) relative improvement in knowledge scores was 30.8% (29.1% - 32.6%) whereas for skills this was 59.8% (58.6% - 60.9%).

### Retention of knowledge and skills

**Fig 1:**
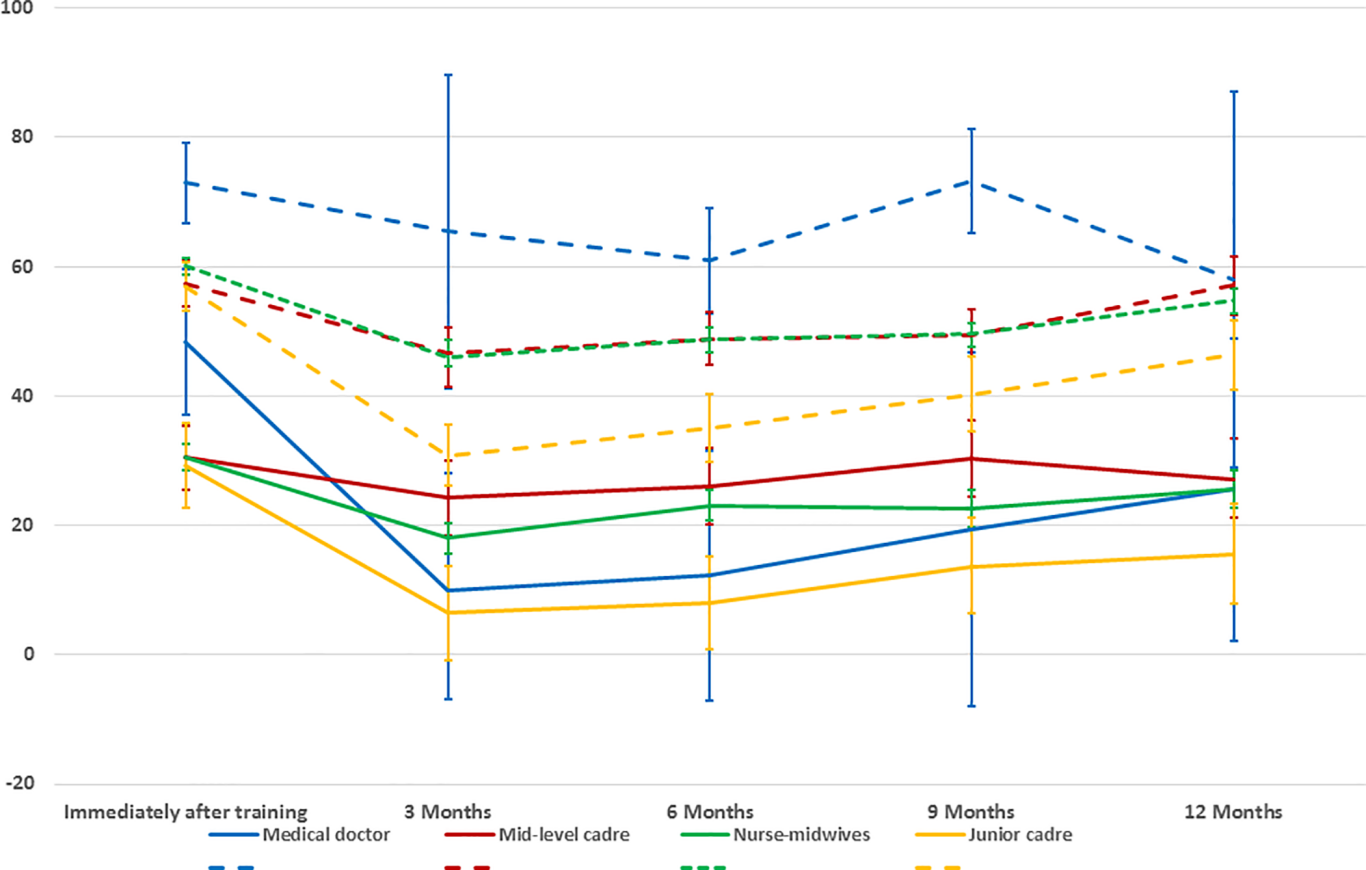
Mean knowledge score change (%) by country over 12 months.

For both knowledge and skills, the relative improvement scores 3 months after the training was completed were lower than immediately after training, with mean (95% CI) relative improvement scores of 17.9% (16.8% - 19.0%) and 43.5% (42.6% - 44.4%) respectively **Fig 1.**

Subsequently, for each cadre, the mean relative improvement scores for knowledge at each subsequent month remained between those immediately post-training and those at 3 months.

The mean relative change in knowledge scores for nurse-midwives (n = 455) was highest (48.6%) immediately after training, lowest (35.1%) at 3-months post training, similar at 6-months (38.7%) and 9 months (37.2%) but not statistically significantly different from immediate post training value at 12-months (42.7%) after the training (**S1 Table**). The pattern was similar for skills, though the values were higher for each assessment (**S2 Table**).

### Factors affecting retention of knowledge and skills

For the NMW cadre mixed effects regression analysis was used to account for potential confounding variables in modelling the relationship between relative change scores and time. For relative changes in both knowledge scores and skills scores over time, the interaction between assessment number and country (p<0.001 in both cases) was statistically significant so each country was analysed separately. The month (p = 0.01), years of experience (p = 0.02), gender (p = 0.04) and number of repeat assessments by country (p<0.001) were statistically significant for skills and were considered for inclusion in the mixed effects regression analysis for relative changes in skills scores for each country. For the combined countries data for knowledge, no other variables were statistically significant. Although not statistically significant in the combined analysis for all countries, month, as well as assessment number, was considered for inclusion in the analyses for each country. For each score no evidence that relative change scores are associated with facility type was detected.

Years of maternity care experience was not statistically significant in the overall analyses for knowledge, years of experience was therefore only considered in each country for skills. Only in Ghana was there evidence of significant variation with years of experience.

For skills, NMW in Ghana who had 13 years or more of maternity care experience, were estimated (mean (95% CI)) to have a relative change score significantly lower on average by 13.8% (5.8%,21.9%) than those with less than 2 years of experience (**S1 Table and S2 Table**).

For each country the analysis of knowledge relative change scores found that either month of assessment (for Ghana, Nigeria and Sierra Leone) or visit number (for Kenya, Malawi, Sierra Leone and Tanzania) had a statistically significant effect on the scores. For month of assessment knowledge relative change scores at months 3, 6, 9 and 12 were usually statistically significantly lower at follow up visits than immediately post training (M0). For assessment visits the reduction was usually greatest at visit 1, with the magnitude of reduction diminishing to visit 4.

For all countries the analysis of skills relative change scores found that visit number had a statistically significant effect on the scores. Tanzania was the only country in which the month of assessment was also statistically significant. For assessment visits skills relative change scores at visits 1, 2, 3 and 4 were usually statistically significantly lower than immediately post training; the reduction was usually greatest at visit 1, with the magnitude of reduction diminishing to visit 4.

Overall, NMW who had completed all four assessments had a higher mean relative change scores for knowledge at the 12-month assessment point compared to NMW who had completed less than three assessments, with a difference of 8.5% (95% CI: -0.3%, 17.4%) however the difference was not statistically significant (p = 0.06). NMWs who had completed four assessments had statistically significantly higher relative change scores for skills at 12 months compared to those who complete less than three assessments 14.9% (95% CI: 7.8%, 22.0%; p<0.001).

## Discussion

### Main findings

A large multi-country study was conducted to assess retention of knowledge and skills of maternity care providers working at healthcare facilities designated to provide Basic or Comprehensive EmOC after they had received standardized training in EmOC.

Analysis of variance using pre-training scores found a significant difference between countries for both knowledge and skills for NMW and mid-level cadre only. At three months mean scores for both knowledge and skills were higher than pre-training scores but lower than those immediately after training was completed. Over a period of 12 months, the mean scores for knowledge and skills at each subsequent assessment remained at levels between those obtained immediately post-training and those at three months.

After correcting for facility type, number of years of experience and number of assessments completed, NMWs who attended the EmOC training and completed all assessment points retained both knowledge and skills close to the levels obtained immediately after training, and, did so for up to one year after the training. Participation in all four repeated assessments and feedback was associated with significantly greater improvements in scores for skills at 12 months (14.9%, 95% CI 7.8%, 22.0%, p<0.001) but this was not the case for knowledge (8.6%, 95% CI -0.3%, 17.4% p = 0.06). Level of healthcare facility at which a healthcare provider worked was used as a proxy measure for workload. This did not affect retention. Years of experience had some effect in Ghana with more than 13 years of experience associated with less retention in skills but not knowledge.

### Strengths and limitations

This is the first multi-country study to assess retention of knowledge and skills after EmOC training for healthcare workers in low- and middle-income countries, measuring retention for up to 12 months following training, accounting for potential confounders and exploring factors that may promote or hinder retention.

We used a before-after study design but accounted for potential confounders, however more powerful design such as stepped wedge design (SWD), will have had appropriate controls without denying maternity care providers the opportunity to participate in training. Available funding and time, limited the use of SWD for this study.

We could not include sufficient numbers of every cadre providing maternity care due to a relatively small number of doctors and mid-cadre staff who were available to participate in this longitudinal study. However, we included NMWs in all countries and this is the main cadre providing frontline maternity services in each of the included settings.

We used the relative change in scores for knowledge and skills to assess improvement or not. This is defined as the percentage change in score relative to the pre-training score. This is a more accurate measure of achievement as healthcare providers with existing high scores for knowledge and skills are not in principle able to achieve as much of an increase as those who have lower scores to begin with [5].

### Interpretation

In principle, all of the cadres included in this study and providing maternity care, received pre-service training to ensure they are competent to provide skilled birth attendance. It could be expected that knowledge and skills levels would be more similar across cadres and countries [12]. However, differences in quality and duration of pre-service medical and midwifery education may account for the some of the difference in pre-training scores observed in this study. Adegoke et al [9] reported that medical doctors in sub-Saharan Africa are generally trained for six years, nurse/midwives and mid-cadre staff for 3–5 years and junior cadre staff are trained for 1–1.5 years. Other factors that were considered as potentially affecting knowledge and skills of maternity care providers include, availability of and participation in regular relevant continuous professional education programmes, years of working in the maternity area and level of healthcare facility at which the healthcare provider works [13]. The latter is related to the workload and type of cases health care providers manage.

Following training, skills were significantly more improved more knowledge, even though pre-training scores for skills were significantly lower than those for knowledge. This is most likely to be because the EmOC training package uses a ‘skills and drills’-type learning approach and is 85% skills-based (hands-on training combined with simulation exercises).

Several previous studies have reported on knowledge and skills retention after EmOC training. These relate to training packages including; all component of EmOC [6,7,14,15] or only some components [16–18]. Study designs used include: RCT [6,17,19] before and after studies [7,14,15] and a systematic review [16] with varying periods of follow-up including; three months [15], six months [6,7,17], nine months [14], 12 months [16] and 24 months [16]. Two studies assessed knowledge only (15,19), skills only [17] and four studies assessed both knowledge and skills [6,7,14,16]. However, to the best of our knowledge, this is the first multi-country study and the first to account for potential confounding factors.

Good retention of acquired knowledge and skills up to nine months has previously been reported [6,7,14,15] after EmOC training but these studies had much smaller sample sizes. Homaifar et al (2013) reported on undergraduate medical students [14]. Two studies reported reduced skills after six months [6,7]. Differences in training methods and content may explain the different results. We used a training package covering all components of EmOC and corrected for facility type, years of experience and number of assessments.

Tang et al [7] noted that lack of practical opportunities (for example, due to low caseload) led to significant reduction of knowledge and skills over time. This was not confirmed in this study because there was no significant change in participants area of work during the study (working in the maternity unit or not) and the level of healthcare facility was not found to be a factor associated with retention of knowledge and skills.

Participation in multiple assessments after initial training resulted in significant improvement in skills but not in knowledge. At each assessment point, participants were given feedback as a group and repeat practice session where possible with learning reinforced as necessary. Retraining at regular intervals has also previously been found to improve skills retention [17,18].

## Conclusion

A standardized training package including all components of EmOC, results in measurably improved knowledge and skills of healthcare workers in low- and middle-income settings which are retained for at least 12 months. Based on the mixed effects regression analysis, short periodic additional ‘skills and drills’ sessions or ‘fire drills’, have the potential to help ensure knowledge and skills are retained over time.

## Supporting information

S1 STROBE Checklist (PDF)

S1 Appendix KSRS questionnaire. (PDF)

S2 Appendix Knowledge and skills test. (PDF)

S3 Appendix AVD practical test. (PDF)

S1 Data Anonymized data KSRS. (XLSX)

S1 Table Fitted models for knowledge relative change scores for nurse-midwives, by country. (DOCX)

S2 Table Fitted models for skills relative change scores for nurse-midwives, by country. (DOCX)
