# Supplementary material for: Retention of knowledge and skills after Emergency Obstetric Care training: A multi-country longitudinal study
Source: PLoS One. 2018 Oct 4;13(10):e0203606. doi: 10.1371/journal.pone.0203606 (PMC6171823; doi:10.1371/journal.pone.0203606)
Supplement: S1 Appendix — (PDF) [file pone.0203606.s002.pdf]

For office use only

C	C	C	D	D	D	D	D	D	A	P	#

Knowledge & Skills Retention Study Questionnaire

Date Today: Day ☐☐ / Month ☐☐ / Year ☐☐

Date Attended LSTM LSS EOC&NC Training: Month ☐☐ / Year ☐☐

Gender: ☐ Male ☐ Female

Age: ☐☐

Participant Number: ☐☐

Country:

State/Province/District:

1. What is the name of the Healthcare Facility where you currently work?:

2. Which department do you work in?:

3a.Have you received any other EmOC or newborn care training in the past 3 months?: ☐ Yes ☐ No

(If no, go to Q3d)

3b.Was the training organised by the health facility where you currently work? ☐ Yes ☐ No

3c.What topics did the course(s) cover?: (Please put a cross X next to all that apply)

- ☐ Adult Resuscitation
- ☐ Newborn Resuscitation
- ☐ Eclampsia Management
- ☐ Haemorrhage
- ☐ Sepsis
- ☐ Managing Obstetric Complications
- ☐ Other *(please specify:)*
- ☐ Vacuum Delivery
- ☐ Manual Removal of Placenta
- ☐ Breech Delivery
- ☐ Management of APH/PPH
- ☐ Managing complications of abortion
- ☐ Completing partographs

3d.How often do you receive EmOC training and/or updates from your facility per year?

- ☐ Never
- ☐ Daily
- ☐ Weekly
- ☐ Monthly
- ☐ Annually
- ☐ Other

Knowledge & Skills Retention Study Questionnaire

4a. On a scale of 1 to 5, how confident do you feel performing the folllowing EOC skills? (please cross x)

Not confident ⟶ Very confident

	1	2	3	4	5
Adult Resuscitation	☐	☐	☐	☐	☐
Newborn Resuscitation	☐	☐	☐	☐	☐
Administering Magnesium Sulphate	☐	☐	☐	☐	☐
Shoulder Dystocia	☐	☐	☐	☐	☐
Manual Removal of Placenta	☐	☐	☐	☐	☐
Breech Delivery	☐	☐	☐	☐	☐
Management of APH	☐	☐	☐	☐	☐
Management of PPH	☐	☐	☐	☐	☐
Vacuum Delivery	☐	☐	☐	☐	☐
Managing Complications of Abortion	☐	☐	☐	☐	☐
Completing Partograph	☐	☐	☐	☐	☐

4b. Approximately how many times have **you** performed the following EmOC skills in the last 3 months?

	0	1-5	6-10	11-20	20+
Adult Resuscitation	☐	☐	☐	☐	☐
Newborn Resuscitation	☐	☐	☐	☐	☐
Administering Magnesium Sulphate	☐	☐	☐	☐	☐
Shoulder Dystocia	☐	☐	☐	☐	☐
Manual Removal of Placenta	☐	☐	☐	☐	☐
Breech Delivery	☐	☐	☐	☐	☐
Management of APH	☐	☐	☐	☐	☐
Management of PPH	☐	☐	☐	☐	☐
Vacuum Delivery	☐	☐	☐	☐	☐
Managing Complications of Abortion	☐	☐	☐	☐	☐
Completing Partograph	☐	☐	☐	☐	☐

4c. What are the 3 main reasons for **non performance** of the skills in the health facility where you currently work?

- ☐ No cases
- ☐ Lack of equipment/supplies/drugs
- ☐ Staff lack confidence
- ☐ National or facility policies do not allow function to be performed
- ☐ Lack of support from senior staff or hospital management
- ☐ Other (please provide details below)

4d. Please provide further details about non performance of skills:
