# Supplementary material for: Retention of knowledge and skills after Emergency Obstetric Care training: A multi-country longitudinal study
Source: PLoS One. 2018 Oct 4;13(10):e0203606. doi: 10.1371/journal.pone.0203606 (PMC6171823; doi:10.1371/journal.pone.0203606)
Supplement: S2 Appendix — (PDF) [file pone.0203606.s003.pdf]

C C C D D D D D D A P P

For office use only

Pre Course Knowledge Assessment

Today's Date: D D / M M / Y Y

Participant number:

This test is intended to sample your opinion on specific issues concerning Emergency Obstetric Care in order for the course organisers to improve the course content.

Communication, Triage and Referral

1. When triaging pregnant women, headache and visual disturbance should be considered an emergency.

   True ☐ False ☐

2. A baby with breathing problems should be reassessed after 2 hours to avoid making an unnecessary referral.

   True ☐ False ☐

3. Records/notes should be sent with a patient who is being transferred in an emergency situation.

   True ☐ False ☐

4. When discussing sensitive issues with a patient, you should ensure that you can not be overheard.

   True ☐ False ☐

5. A patient in prolonged labour needs to be transferred to a CEOC as soon as she crosses the action line on the partograph.

   True ☐ False ☐

Maternal and Newborn Resuscitation

1. Neonatal resuscitation is usually best managed with a bag and mask.

   True ☐ False ☐

2. When assessing the airway you shold look, listen and feel for breathing.

   True ☐ False ☐

3. The Great Saphenous vein is about 1 finger anterior and superior to the medial malleolus.

   True ☐ False ☐

4. If an unconcisous patient is not breathing, an endotracheal tube should be inserted before continuing resuscitation.

   True ☐ False ☐

5. Careful suctioning should only be carried out if there is thick meconium in a floppy baby.

   True ☐ False ☐

Shock and the Unconscious Patient

1. Unsafe abortion is a frequent cause of septic shock.

True ☐ False ☐

2. Shock is a life threatening condition.

True ☐ False ☐

3. It is advisable to use a spatula or tongue depressor in a woman with eclamptic fits to prevent her biting her tongue.

4. True ☐ False ☐

The first thing to do when you encounter an unconscious person is to quickly check

5. the pulse so as to know whether to start CPR.

True ☐ False ☐

A decreased level of consciousness is a marker of lack of oxygen to the brain.

True ☐ False ☐

(Pre) Eclampsia

1. Magnesium sulphate should be used for lowering blood pressure.

True ☐ False ☐

2. Korotkoff phase 5 should be used when measuring diastolic blood pressure.

True ☐ False ☐

3. When giving magnesium sulphate, check the woman's pulse, BP and respiration rate hourly.

True ☐ False ☐

4. Nifedipine and labetalol are safe to give to pregnant women.

True ☐ False ☐

5. When a woman is receiving magnesium sulphate, if her respiration rate drops below 16 breaths/min, stop magnesium and give calcium gluconate.

True ☐ False ☐

Obstetric Haemorrhage

1. Haemorrhage is the most common cause of maternal mortality worldwide.

True ☐ False ☐

2. Coagulation failure is likely to occur in cases of sepsis and placental abruption.

True ☐ False ☐

3. In cases of obstetric haemorrhage, one large bore intravenous cannula is usually sufficient for resuscitation.

True ☐ False ☐

4. Emptying the bladder is an important part of the management of postpartum haemorrhage.

True ☐ False ☐

5. Antepartum haemorrhage associated with a tender uterus is likely to be caused by placenta praevia.

True ☐ False ☐

Obstructed Labour

1. Slow progress in labour may be due to one of the 3 P's

   True ☐ False ☐

2. Oxytocin may be used to suppress labour prior to transfer.

   True ☐ False ☐

3. A multigravida at term having 2 contractions lasting 20 seconds, membranes intact and a cervical dilation of 2-3cm is in active labour.

   True ☐ False ☐

4. A woman is brought to your health facility with a history of 16 hours of labour, the correct diagnosis is obstructed labour.

   True ☐ False ☐

5. The commonest cause of secondary arrest of labour is occipito-transverse position.

   True ☐ False ☐

Obstetric Emergencies

1. The most effective way of initiating delivery of shoulder dystocia is to apply fundal pressure and pull.

   True ☐ False ☐

2. A breech presentation associated with any other complication is an indication for Caesarean Section.

   True ☐ False ☐

3. Repositioning of an inverted uterus should be done immediately.

   True ☐ False ☐

4. In shoulder dystocia, it is usually easier to deliver the anterior arm than the posterior arm.

   True ☐ False ☐

5. When doing a breech delivery, you should hold the baby around the hips and stomach to give a firm grip during delivery.

   True ☐ False ☐

Pregnancy related sepsis and unsafe Abortion

1. Patients for MVA do not need painkillers before the procedure.

   True ☐ False ☐

2. Counselling on contaceptives and family planning is an essential element of post abortion care.

   True ☐ False ☐

3. For paracervical block in MVA, 1ml of 0.5% lignocaine solution with adrenaline should be injected into ther cervical skin at the 3, 5, 7 and 9 o'clock positions.

   True ☐ False ☐

4. Usually in MVA, the use of 0.5% chlorine solution for decontaminating equipment can be omitted

   True ☐ False ☐

5. During MVA, complete uterine evacuation can be recognized by gritty sensation, reduced bleeding and air bubbles in the cannulae.

   True ☐ False ☐
