# Supplementary material for: Retention of knowledge and skills after Emergency Obstetric Care training: A multi-country longitudinal study
Source: PLoS One. 2018 Oct 4;13(10):e0203606. doi: 10.1371/journal.pone.0203606 (PMC6171823; doi:10.1371/journal.pone.0203606)
Supplement: S3 Appendix — (PDF) [file pone.0203606.s004.pdf]

For office use only

C	C	C	D	D	D	D	D	D	A

Assisted Vaginal Delivery

Room: ____ **Todays date:** __ / __ / __ **Facilitator:** ____

Pre Test ☐ **Post Test** ☐

Equipment Needed: Lucy & mum, latex gloves, lubricant, vacuum extractor

Facilitators please note: marks should be as whole numbers, not fractions.

Scenario: A 30 year old, para 2, at 37 weeks gestation in second stage of labour.

	Mark	Participant Number			
**Please outline 4 indications for AVD.** Any 4 of the following in 2nd stage: prolonged 2nd stage; fetal distress; maternal exhaustion; placental abruption; cord prolapse.	**4**				
**Can you tell me 4 pre-requisites for AVD.** Any 4 of the following: Vertex position; position known with certainty; full cervical dilatation; fetal head at spines or lower; ruptured membranes; adequate analgesia; empty bladder; maternal consent and co-operation.	**4**				
**Please demonstrate how you would deliver the baby using vacuum extraction.** Correct identification of position, cup application at flexion point, and exclude maternal tissue.	**6**				
Pull in the correct axis, pull during contractions, remove cup after head is delivered.	**3**				
**Can you tell me 3 possible complications of vacuum extraction.** Maternal: Perineal/vaginal lacerations/injuries. Fetal: scalp abrasions/lacerations, cephalhaematoma.	**3**				
**Total Score:**	**20**				

Faculty Comments
