# Supplementary material for: Retention of knowledge and skills after Emergency Obstetric Care training: A multi-country longitudinal study
Source: PLoS One. 2018 Oct 4;13(10):e0203606. doi: 10.1371/journal.pone.0203606 (PMC6171823; doi:10.1371/journal.pone.0203606)
Supplement: S1 Table — (DOCX) [file pone.0203606.s006.docx]

**Supplementary Table 1: Fitted models for knowledge relative change scores for nurse-midwives, by country**

| Covariate / Risk factor | **Country** | | | | | |
| --- | --- | --- | --- | --- | --- | --- |
|  | Ghana  (n^a^=114) | Kenya  (n^a^=97) | Malawi  (n^a^=63) | Nigeria  (n^a^=95) | Sierra Leone  (n^a^=64) | Tanzania  (n^a^=55) |
| Constant | **107.2 (93,122)** | **101.7 (72.8,130.6)** | **71.3 (45.1,97.6)** | **109.4 (88.0,130.9)** | **93.0 (74.7,111.3)** | **76.0 (58.1,94.0)** |
| Pre-training | **-1.19 (-1.41,-0.97)** | **-0.98 (-1.38,-0.57)** | **-0.63 (-1.01,-0.25)** | **-1.17 (-1.49,-0.84)** | **-1.04 (-1.32,-0.76)** | **-0.77 (-1.06,-0.48)** |
| Month of assessment | | | | | | |
| M3 vs M0 | **-8.5 (-13.8,-3.2)** |  |  | **-12.2 (-19.6,-4.8)** | **-28.7 (-35.8,-21.5)** |  |
| M6 vs M0 | **-6.4 (-11.6,-1.2)** | P=0.93 | P=0.06 | **-12.4 (-20.4,-4.4)** | -10.7 (-25.4,4.1) | P=0.26 |
| M9 vs M0 | **-8.6 (-13.8,-3.3)** |  |  | -5.3 (-13.7,3.0) | **-30.8 (-42.9,-18.7)** |  |
| M12 vs M0 | -0.9 (-6.3,4.6) |  |  | **-21.2(-30.2,-12.2)** | **-31.3 (-46.1,-16.5)** |  |
| Assessment visit number | | | | | | |
| V1 vs V0 |  | **-12.3 (-17.1,-7.6)** | -7.1 (-14.2,0.1) |  | 0^a^ | **-8.5 (-16.0,-1.2)** |
| V2 vs V0 | P=0.70 | **-5.6 (-10.8,-0.4)** | -3.4 (-10.5,3.8) | P=0.35 | -2.1 (-15.6,11.4) | -6.4 (-13.8,1.1) |
| V3 vs V0 |  | -5.5 (-11.3,0.3) | 4.5 (-2.8,11.8) |  | 10.7 (-2.1,23.4) | **-8.8 (-16.6.-1.0)** |
| V4 vs V0 |  | **9.1 (1.9,16.3)** | -6.9 (-14.4,0.5) |  | **27.8 (11.6,44.0)** | -1.1 (-9.9,7.6.3) |

# emboldened estimates indicate that the estimated effect was significantly different from 0, using a Wald test.

a when both month and visit are included in the model V1 is not estimated as there is collinearity
