# Supplementary material for: Retention of knowledge and skills after Emergency Obstetric Care training: A multi-country longitudinal study
Source: PLoS One. 2018 Oct 4;13(10):e0203606. doi: 10.1371/journal.pone.0203606 (PMC6171823; doi:10.1371/journal.pone.0203606)
Supplement: S2 Table — (DOCX) [file pone.0203606.s007.docx]

**Supplementary Table 2: Fitted models for skills relative change scores for nurse-midwives, by country**

| Covariate / Risk factor | **Country** | | | | | | |
| --- | --- | --- | --- | --- | --- | --- | --- |
|  | Ghana  (n^a^=108/114) | Kenya  (n=97) | Malawi  (n=63) | Nigeria  (n=95) | Sierra Leone  (n=64) | Tanzania  (n=55) | |
| Constant | **70.0 (54.9,85.2)** | **52.8 (38.3,67.3)** | **97.2 (75.7,118.7)** | **62.8 (48.3,77.3)** | **56.2 (44.2,68.2)** | **86.3 (71.5,101.2)** | |
| Pre-training | -0.06 (-0.31,0.18) | 0.23 (-0.07,0.53) | **-0.59 (-0.94,-0.24)** | **-0.03 (-0.36,0.30)** | -0.05 (-0.31,0.20) | **-0.52(-0.82,-0.21)** | |
| Month of assessment | | | | | | |  |
| M3 vs M0 |  |  |  |  |  | **-15.2 (-18.9,-11.6)** | |
| M6 vs M0 | P=0.39 | P=0.11 | P=0.97 | P=0.30 | P=0.38 | **-34.0 (-45.2,-22.9)** | |
| M9 vs M0 |  |  |  |  |  | **-32.0 (-46.6,-17.4)** | |
| M12 vs M0 |  |  |  |  |  | **-35.1 (-55.9,-14.3)** | |
| Assessment visit number | | | | | | |  |
| V1 vs V0 | **-10.7 (-13.4,-7.9)** | **-6.6 (-9.4,-3.8)** | **-18.2 (-22.9,-13.5)** | **-15.5 (-19.1,-11.9)** | **-29.5 (-33.1,-25.9)** | 0^b^ | |
| V2 vs V0 | **-6.4 (-9.5,-3.3)** | **-4.8 (-7.9,-1.7)** | **-13.6 (-18.4,-8.8)** | **-11.6 (-15.5,-7.7)** | **-19.9 (-24.0,-15.8)** | **19.0 (7.7,30.2)** | |
| V3 vs V0 | -2.6 (-6.0,0.7) | **-4.8 (-8.2,-1.4)** | **-16.9 (-22.0,-11.8)** | **-6.0 (-10.2,-1.8)** | **-17.8 (-22.8,-12.7)** | **22.3 (7.2,37.4)** | |
| V4 vs V0 | **-4.5 (-8.6,-0.4)** | -1.7 (-5.8,2.5) | -2.7 (-8.1,2.7) | -2.0 (-7.2,3.2) | -12.2 (-18.3,-6.1) | **29.8 (8.3,51.4)** | |
| Years of experience | | | | | | | |
| Yrs 2-3 vs <2 | -3.0 (-11.5,5.4) |  |  |  |  |  | |
| 4-6 vs <2 | -7.9 (-16.5,0.8) | P=0.15 | P=0.12 | P=0.09 | P=0.83 | P=0.58 | |
| 7-12 vs <2 | **-9.7 (-17.7,-1.8)** |  |  |  |  |  | |
| 13+ vs <2 | **-13.8 (-21.9,-5.8)** |  |  |  |  |  | |

# emboldened estimates indicate that the estimated effect was significantly different from 0, using a Wald test

a number included in multivariable models involving years of experience / number included in the fitted model without these variables; when years of experience was significant

b when both month and visit are included in the model V1 is not estimated as there is collinearity
